# The Citrus Flavanone Naringenin Protects Myocardial Cells against Age-Associated Damage

**DOI:** 10.1155/2017/9536148

**Published:** 2017-03-12

**Authors:** Eleonora Da Pozzo, Barbara Costa, Chiara Cavallini, Lara Testai, Alma Martelli, Vincenzo Calderone, Claudia Martini

**Affiliations:** ^1^Department of Pharmacy, University of Pisa, Pisa, Italy; ^2^Interdepartmental Research Center “Nutraceuticals and Food for Health”, University of Pisa, Pisa, Italy

## Abstract

In recent years, the health-promoting effects of the citrus flavanone naringenin have been examined. The results have provided evidence for the modulation of some key mechanisms involved in cellular damage by this compound. In particular, naringenin has been revealed to have protective properties such as an antioxidant effect in cardiometabolic disorders. Very recently, beneficial effects of naringenin have been demonstrated in old rats. Because aging has been demonstrated to be directly related to the occurrence of cardiac disorders, in the present study, the ability of naringenin to prevent cardiac cell senescence was investigated. For this purpose, a cellular model of senescent myocardial cells was set up and evaluated using colorimetric, fluorimetric, and immunometric techniques. Relevant cellular senescence markers, such as X-gal staining, cell cycle regulator levels, and the percentage of cell cycle-arrested cells, were found to be reduced in the presence of naringenin. In addition, cardiac markers of aging-induced damage, including radical oxidative species levels, mitochondrial metabolic activity, mitochondrial calcium buffer capacity, and estrogenic signaling functions, were also modulated by the compound. These results suggested that naringenin has antiaging effects on myocardial cells.

## 1. Introduction

Naringenin (Nar), a bitter flavanone mainly present in citrus fruits and tomatoes, is a common component of the human diet. Recently, this compound has received considerable attention for its health-promoting and disease-preventing effects, and interest in its pharmaceutical and nutritional effects has also increased [1]. Among the many biological targets and molecular mechanisms that underlie its beneficial activities, its antioxidant actions are the best characterized. Nar is a free radical scavenger, a metal ion chelator, and an activator of the antioxidant enzyme defense [1, 2]. In addition, Nar stimulates the mitochondrial calcium-dependent potassium channel (mitoBKCa), which causes an influx of potassium ions, a mild depolarization, and a decrease in the mitochondrial matrix calcium uptake, all of which contribute to stabilizing the mitochondria during cellular damage [3, 4]. Furthermore, Nar has been demonstrated to bind to estrogen receptors [5–8] and shows bidirectional adjusting effects [9]. On the basis of these activities, it has been suggested that this compound could be useful as a dietary component during cardiometabolic disorders, also associated with estrogen deficiency [10, 11]. The cardioprotective effects of Nar are well documented in the healthy state, in myocardial infarction, and in daunorubicin-, doxorubicin-, and high-glucose-induced cardiotoxicity [2, 12–17]. A very recent publication has also provided evidence that Nar has beneficial effects in the livers of old rats [18]. Because aging has been demonstrated to be directly related to the occurrence of cardiac disorders, together, the data have prompted us to investigate the effects of Nar in a cellular model of aged myocardial cells. To the best of our knowledge, the effect of Nar in cardiac cell senescence has not yet been studied.

An in vitro model of premature myocardial senescence was established as previously reported [19]. The studies were carried out in the absence and in the presence of Nar. Various cellular senescence hallmarks (the percentage of X-gal staining cells, the mRNA levels of the p16 and p21 cell cycle regulators, and the percentage of cell cycle-arrested cells) were investigated. In addition, some pathways that are typically altered during cardiac aging-induced damage, including the generation of radical oxidative species, the mitochondrial metabolic activity, the modulation of the mitochondrial calcium buffering capacity, and the regulation of estradiol and estrogen-regulated gene expression, were investigated [20–22].

The results demonstrated that Nar exerts effective antiaging properties in myocardial cells.

## 2. Materials and Methods

### 2.1. Chemicals

**Figure 1:**
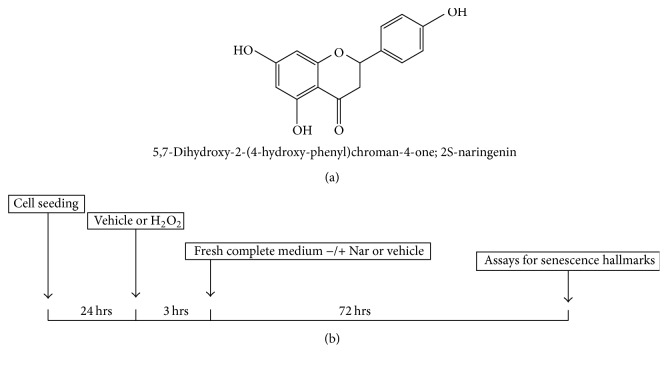
(a) The structure of Nar. (b) Schematic depiction of the cell culture treatment protocol.

Nar (5,7-dihydroxy-2-(4-hydroxy-phenyl)chroman-4-one) (Figure 1(a)) and paxilline were purchased from Sigma-Aldrich (Milan, Italy). They were dissolved (10^−2 ^M) in DMSO and further diluted in cell culture medium. All other reagents were purchased from standard sources.

### 2.2. Cell Culture

H9c2 cells (ATTC, Manassas, VA, USA), a subclonal cell line that was derived from embryonic rat hearts [23], were cultured in DMEM (Sigma-Aldrich, Milan, Italy), supplemented with 10% fetal bovine serum (FBS, Sigma-Aldrich, Milan, Italy), 100 units/mL penicillin, and 100 mg/mL streptomycin in tissue culture flasks at 37°C in a humidified atmosphere of 5% CO_2_.

### 2.3. H9c2 Cell Senescence Model

H9c2 cell senescence was induced by the exogenous oxidative insult H_2_O_2_ as previously reported [19]. Briefly, the cells were seeded at a density of 10 × 10^3^ cells/cm^2^. After 24 h to allow cell attachment, the cells were treated with vehicle (water) or various concentrations of hydrogen peroxide (H_2_O_2_) (5–100 *μ*M) for 3 hours and subsequently cultured for 3 days, after which the senescence hallmarks were determined (Figure 1(b)). In parallel, low-micromolar concentrations (4 and 40 *μ*M) of Nar or vehicle (0.1% DMSO) were added to the cells immediately after the senescence insult and maintained in the medium for 3 days.

### 2.4. Senescence-Associated *β*-Galactosidase (sa-*β*-Gal) Staining

To evaluate the percentage of senescent cells 3 days after the H_2_O_2_ insult, staining for the senescence marker sa-*β*-gal was performed as previously reported [25]. Briefly, the treated cells were fixed in p-formaldehyde and incubated in staining solution for 16 hours (37°C, dry incubator). The cells were then washed in PBS (1x), and images of randomly selected light microscopic fields were captured (5 fields per well, 100x). Both blue (senescent) and uncolored (nonsenescent) cells were counted using ImageJ (ImageJ Software, version 1.41, USA).

### 2.5. RNA Extraction and Real-Time PCR Analysis

H_2_O_2_-injured H9c2 cells were collected, and total RNA was extracted using the RNeasy® Mini Kit (Qiagen, Hilden, Germany) according to the manufacturer's instructions. cDNA synthesis was performed (500 ng of RNA) using the i-Script cDNA synthesis kit (BioRad, Hercules, USA). Real-time RT-PCR reactions (25 *μ*L, Fluocycle® II SYBR® [Euroclone, Milan, Italy], 1.5 *μ*L of 10 *μ*M forward and reverse primers for the p16 and p21 cell cycle regulators (Sigma-Aldrich, Milan, Italy), 3 *μ*L cDNA, and 19 *μ*L H_2_O) were performed for 38 cycles using the following temperature profiles: 94°C for 1 minute (initial denaturation); 55–59°C for 30 seconds (annealing); and 72°C for 1 second (extension).

Furthermore, the same real-time RT-PCR reactions were performed to assess the expression of two estrogen-regulated genes; estrogen receptor *β* (ER *β*) and vitamin D receptor (VDR) were studied as indicators of estrogenic activity [26]. The list of primers used in the study was shown in Table 1.

### 2.6. Cell Cycle Analysis

The percentages of H_2_O_2_-injured cells in the various cell phases were determined using the Muse™ Cell Cycle reagent (Merck KGaA, Darmstadt, Germany). Briefly, senescent adherent cells were collected and centrifuged at 300 ×g for 5 minutes. The pellet was washed with PBS and suspended in 100 *μ*L of PBS and then slowly added to 1 mL of ice-cold 70% ethanol and maintained overnight at −20°C. Then, an aliquot of the cell suspension (2 × 10^5^ cells) was centrifuged, washed with PBS, suspended in the nuclear DNA stain, propidium iodide, and analyzed [27].

### 2.7. ROS Production

The ROS generation was assessed using the fluorogenic probe DCFH_2_-DA (Molecular Probes, Invitrogen) [19, 28]. Briefly, injured H9c2 cells, treated with Nar or vehicle, were washed in PBS/10 mM glucose (loading buffer) and loaded with 8 *μ*M DCFH_2_-DA for 30 min in the dark (37°C). The cells were then washed and incubated in loading buffer. FDA fluorescence was estimated using a plate reader with wavelengths of 485 nm (excitation) and 520 nm (emission) (Wallac, Victor 2, 1420 multilabel counter, PerkinElmer) 30 minutes later. Then, after washing with PBS/10 mM glucose, the cells were incubated with crystal violet for 30 min at room temperature. After extensive washing, a solution of 1% SDS was added to each well, the plates were mechanically shaken for 1 h, and the absorbance at 595 nm was determined. The DCFH_2_-DA fluorescence values were normalized to the cell content of each well as indicated by the crystal violet assay.

### 2.8. Comet Assay

The DNA damage was assessed using the Comet assay as previously reported [29]. Briefly, microscope slides were coated with 0.5% normal-melting-point-agarose (NMA) in calcium- and magnesium-free PBS, covered with a coverslip, and kept at 4°C in a humid box until use. H9c2 cell suspensions (5 × 10^3^ cells) were added to 1% low-melting-point agarose (LMPA, 35°C). A volume of the LMPA-embedded sample was layered over the NMA layer. After the agarose solidified, a final layer of 1% LMPA was added. Following the solidification of this layer of agarose, the coverslips were removed and the slides were treated with lysing solution (10% DMSO, 1% TRITON X-100, 2.5 M NaCl, 100 mM EDTA, 10 mM TRIS, and 1% sodium sarcosinate, pH 10) for 1 h at 4°C in the dark. The slides were rinsed in distilled water and placed in a horizontal gel electrophoresis apparatus containing 75 mM NaOH and 1 mM EDTA, pH 12, for 20 min. Electrophoresis was carried out at 25 V and 300 mA for 10 min. The slides were then removed and incubated in neutralizing solution (0.4 M TRIS, pH 7.5, 5 min) three times. The slides were drained, stained with ethidium bromide, and observed using a fluorescence microscope (400x). The amount of damaged DNA (migrated in the tail, Comet) was expressed as percent of total fluorescence for each nucleus. To calculate the distribution of DNA damage, the comets were classified into five categories according to the percent of the DNA in the tail: class I: 0–6% (no damage); class II: 6.1–17% (low damage); class III: 17.1–35% (moderate damage); class IV: 35.1–60% (high damage); and class V: 60.1–100% (extreme damage) [30].

### 2.9. Mitochondrial Metabolic Activity

Briefly, the mitochondrial metabolic activity of senescent H9c2 cells that had been treated with Nar or vehicle was determined using the [3-(4,5-dimethylthiazol-2-yl)-5-(3-carboxymethoxyphenol)-2-(4-sulfophenyl)-2H-tetrazolium, inner salt] (MTS) assay according to manufacturer's instructions (Promega, Milano, Italy) [31]. This tetrazolium dye can be reduced by the metabolic reducing agents NADH and NADPH to a water-soluble formazan salt; the amount of formazan produced is considered to be a marker of the oxidative metabolic activity index [32]. MTS reagent was added to the PIGA ligand-treated cells, and the colorimetric MTS conversion was quantified after 2 h by measuring the absorbance at 490 nm using a microplate reader (WallacVictor 2, 1420 Multilabel Counter, Perkin Elmer, USA).

### 2.10. Calcium Retention Capacity Measurement

The calcium retention capacity (CRC) was determined as previously described [33] using Calcium Green-5N (*λ*_ex_ = 505  nm, *λ*_em_ = 535 nm), a low affinity nonmembrane-permeant probe that increases its fluorescence emission upon binding Ca^2+^. Senescent H9c2 cells (2 × 10^6^ cells) were suspended in CRC buffer (250 mM sucrose, 1 mM Pi-Tris, and 10 mM MOPS-Tris, pH 7.4), permeabilized with digitonin (40 *μ*M, for 5 min, 0°C), and incubated with the respiratory substrate 5 mM succinate and 0.25 *μ*M Calcium Green-5 N (25°C). Ca^2+^ pulses (10 nmoles) were added at 3-minute intervals until the onset of the permeability transition, in the presence or absence of Nar. To evaluate the involvement of mitoBK_Ca_ channels in the CRC modulation, the selective mitoBK_Ca_ blocker paxilline was applied. The fluorescence was measured using a microplate fluorimeter equipped with thermostatic control (Victor Wallac 2, Perkin Elmer, CA, USA). The results are presented as the sample CRC normalized to the CRC of the control (CRC_0_).

### 2.11. Estradiol Levels in the Conditioned Cell Medium

The amounts of estradiol in the conditioned medium of senescent H9c2 cells that had been treated with Nar or vehicle were measured using a competitive EIA assay according to the manufacturer's instructions (Estradiol EIA Kit, Cayman Chemical, Ann Arbor, MI, USA). The absorbance data obtained from the standard curve and the cell supernatants were plotted as logit B/B_0_ versus log concentration and fit using a linear model. To monitor the possible interference of Nar in the assay, standard curves in the presence of Nar concentrations were also performed.

### 2.12. Statistical Analyses

The nonlinear multipurpose curve-fitting program GraphPad Prism (GraphPad Software Inc., San Diego, CA) was used for the data analysis and graphic presentations. All results are presented as the means ± standard errors of the means (SEM) of data for triplicate samples and are representative of three different experiments. The statistical analyses were performed using a *t*-test or one-way analysis of variance (ANOVA) with Bonferroni's corrected *t*-test for post hoc pair-wise comparisons. *p* < 0.05 was considered statistically significant.

## 3. Results and Discussion

Several epidemiological studies have suggested an inverse correlation between aging-associated disorders, in particular cardiovascular diseases, and the intake of citrus fruits [34, 35]. Grapefruit, bergamot, and sour oranges contain high amounts of the flavanone-7-O-glycoside naringin, which is metabolized by the human gut microflora to the aglycone Nar [36] (Figure 1(a)). It has been estimated that the mean peak of the plasma levels of Nar reaches micromolar concentrations following consumption of a serving of citrus fruit [37–39]. Various pharmacological effects including antimutagenic, antiatherogenic, antihypertensive, and anti-inflammatory activities have been reported for Nar [40–44]. Recently, the prevention of cell death by flavanone has been studied in H9c2 myocardial cells that were injured by exposure to a high concentration of glucose or to daunorubicin. The results have suggested that Nar significantly ameliorates the drug-induced toxicity by inhibiting apoptosis [1, 2]. A very recent publication has demonstrated that Nar has the ability to improve the antioxidant status and membrane phospholipid composition in old rats [18]. These data prompted us to investigate the effects of Nar in a cellular model of aged myocardial cells.

### 3.1. Senescence-Associated *β*-Galactosidase Staining and Cell Cycle Investigations

In the present study, the cellular senescence model that was used to assess the cardioprotective action of Nar was established with H9c2 cells that were exposed to H_2_O_2_ to induce senescence. Despite their embryonic origin, H9c2 cells are similar to normal primary cardiomyocytes with respect to their energy metabolism and have been successfully used as an in vitro model for studies of myocardial pathophysiology, including the aging processes [19, 45–49].

Myocardial cellular senescence was triggered by exposing the cells to H_2_O_2_ as previously reported [19], and the presence of sa-*β*-gal product was monitored as a marker of cellular senescence. To establish the effective dose of H_2_O_2_, H9c2 cells were exposed to a range of concentrations of H_2_O_2_ (from 5 *μ*M to 100 *μ*M) for 3 hours and subsequently cultured with fresh complete medium for 72 h (Figure 1(b)).

**Figure 2:**
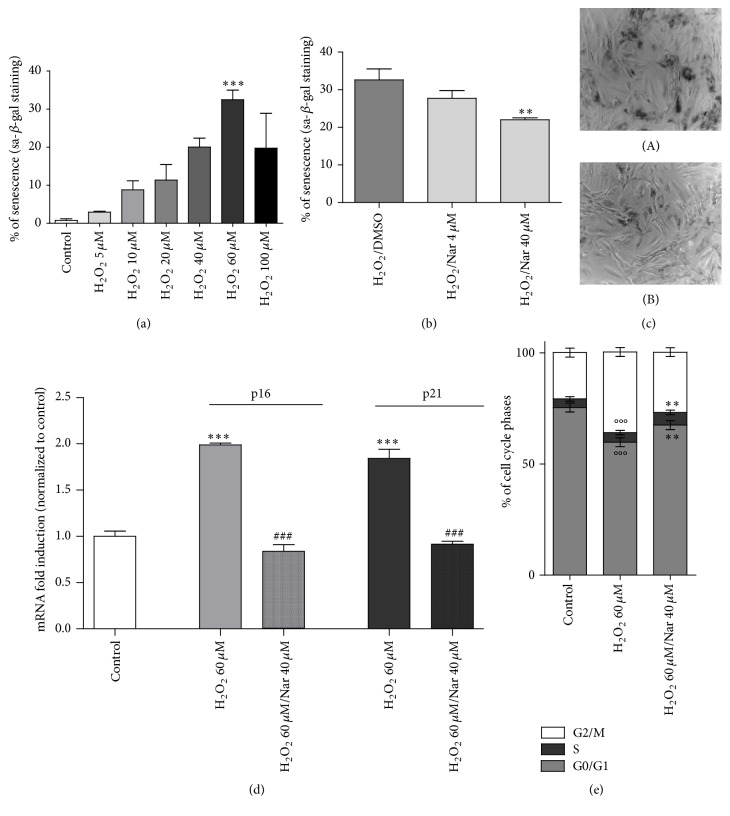
Nar effects on H9c2 cell senescence hallmarks. (a) Senescence-associated b-galactosidase staining. The data are shown as the percentages of *β*-galactosidase-positive cells. Each bar represents the mean ± SEM of three replicates from three independent experiments. ^*∗∗∗*^*p* < 0.01 versus the control group; (b) the data are shown as percentage of *β*-galactosidase-positive cells. Each bar represents the mean ± SEM of three replicates from three independent experiments. ^*∗∗*^*p* < 0.01 versus the H_2_O_2_-treated cells. (c) Representative phase contrast photomicrographs of treated cells; (A) H_2_O_2_-treated cells; (B) 40 *μ*M Nar-cotreated cells. (d, e) Cell cycle arrest machinery. (d) p16 and p21 mRNA fold induction. Each bar represents the mean ± SEM of three replicates from three independent experiments. ^*∗∗∗*^*p* < 0.001 versus the control group; ^###^*p* < 0.001 versus the H_2_O_2_-treated group. (e) Cell cycle phases. Each bar represents the mean ± SEM of three replicates from three independent experiments. °°°*p* < 0.001 versus the control group; ^*∗∗*^*p* < 0.01 versus the H_2_O_2_-treated group.

The presence of sa-*β*-gal product after H_2_O_2_ incubations was examined (Figure 2). A concentration-dependent increase in the sa-*β*-gal activity was observed in the H_2_O_2_-exposed cells; the 60 *μ*M H_2_O_2_ concentration effectively induced significant staining for sa-*β*-gal (*p* < 0.01, Figure 2(a)). A higher dose of H_2_O_2_ (100 *μ*M) did not further increase the senescence marker, probably due to its cytotoxic effect (data not shown). Thus, the 60 *μ*M concentration of H_2_O_2_ was established as the effective drug concentration (*E*_max_) for use in the cell treatment protocol as shown in Figure 1.

The cell cycle regulators p16 and p21, which are mainly activated during the senescence process, and the percentage of cells in the various cell cycle phases were then evaluated. The real-time RT-PCR results showed that the H_2_O_2_ cell treatments significantly increased the levels of p16 and p21 mRNA (*p* < 0.01 and *p* < 0.001, Figure 2(d)). Consistent with these results, the cell cycle analysis demonstrated that the cell cycle was arrested in G2/M phase following H_2_O_2_ treatment (*p* < 0.001, Figure 2(e)). All of these results are consistent with the literature [19, 50–54]. Specifically, previous studies have demonstrated that the number of senescent cells as indicated by X-gal staining and the p21 cell cycle regulator are increased in a dose-dependent manner by treatment with H_2_O_2_ and that the cell cycle is arrested at the G1 and G2/M phases [19, 53]. Furthermore, it has been demonstrated that challenge of H9c2 cells with H_2_O_2_ induces a significant increase in the intracellular ROS level and in the olive tail moment, which indicates an increase in DNA damage [19].

Therefore, H9c2 cells were challenged with 60 *μ*M H_2_O_2_ for 3 hours and then cultured with fresh complete medium containing various concentrations of Nar. Senescence hallmarks were assessed 72 h later (Figure 2(b)).

Treatment with 40 *μ*M Nar significantly protected the cells from the senescence-inducing insult; specifically, it decreased the number of sa-*β*-gal positive cells (*p* < 0.01, Figures 2(b) and 2(c)). In contrast, treatment with 4 *μ*M Nar failed to prevent the development of cellular senescence, although a trend for a reduction in the number of senescent cells was evident (Figure 2(b)). Thus, the 40 *μ*M concentration was selected for subsequent assays. Notably, previous human pharmacokinetic analyses have shown that Nar, following a single oral administration of 135 mg (equal to 1 or 2 glasses of grapefruit juice), is rapidly absorbed and its concentration in plasma reaches a peak of approximately 10 *μ*M in 3.5 hours [27, 55]. Furthermore, Nar cotreatment abolished the increase in p16 and p21 mRNA due to H_2_O_2_ (*p* < 0.01 and *p* < 0.001, Figure 2(d)) and reduced the cell cycle arrest in G2/M (*p* < 0.01, Figure 2(e)).

Collectively, the present data showed for the first time that Nar treatment counteracts the manifestation of the senescent hallmarks in H_2_O_2_-treated cells, which suggests that it has the ability to prevent the prematurely induced senescence in H9c2 cells. In a very recent review, Nar was identified as an effective antiaging ingredient among the traditional Chinese medicine natural products [56]. This hypothesis has been proposed based on the activity of Nar to increase glucose uptake in skeletal muscle cells through activation of AMPK [57]. In addition, the recent data obtained using old rats have confirmed this assumption. Indeed, flavanone administration to old male rats was able to modulate the activity of antioxidant enzymes including catalase, superoxide dismutase 1, and glutathione reductase. All of these enzymes significantly decrease during aging, and their activities were found to be increased in flavanone-fed old rats compared to vehicle-treated animals. Furthermore, thiobarbituric acid reactive substances (TBARS) were significantly decreased by Nar administration, and the membrane phospholipid composition was improved in favor of *ω*-3 PUFA and the *ω*-6/*ω*-3 PUFA ratio [18].

### 3.2. Nar Reduced ROS Generation and DNA Damage

**Figure 3:**
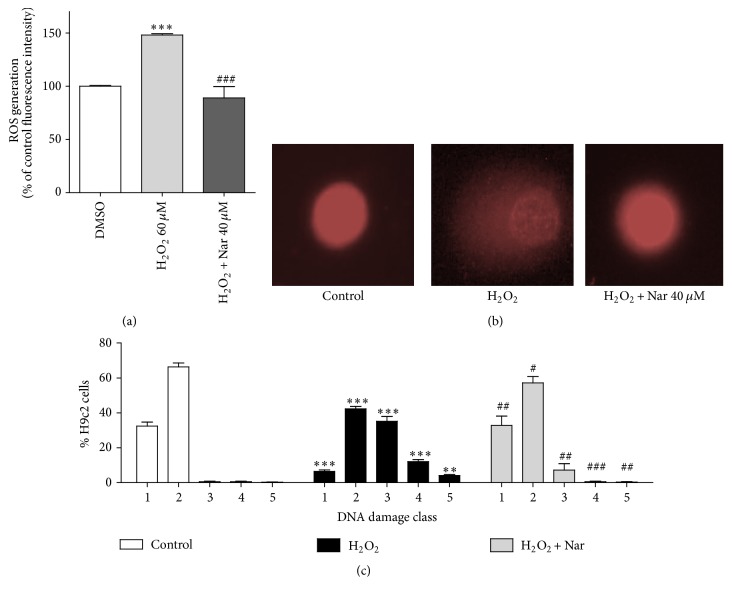
ROS production and DNA damage. (a) ROS generation in H9c2 cells. Each bar represents the mean ± SEM of three replicates from three independent experiments. ^*∗∗∗*^*p* < 0.001 versus the control cells, and ^###^*p* < 0.001 versus the H_2_O_2_-treated cells. (b) Representative photomicrographs of cells assessed using the Comet assay. (c) DNA damage was calculated as classes of DNA damage, as described in Section 2.8. ^*∗∗∗*^*p* < 0.001 and ^*∗∗*^*p* < 0.01 versus control; ^###^*p* < 0.001, ^##^*p* < 0.01, and ^#^*p* < 0.05 versus the H_2_O_2_-treated cells.

In this context, among the pathways typically impaired during cardiac aging-associated damage, the cellular mechanisms associated with the generation of radical oxidative species (ROS) generation and alterations in DNA integrity are frequently altered [58, 59]. These parameters were therefore assessed in our model. As shown in Figure 3, H_2_O_2_ treatment caused a significant increase in ROS generation (*p* < 0.001, Figure 3(a)), and cotreatment of the cells with Nar resulted in maintenance of the ROS levels as those of the control (*p* < 0.001 versus cells treated with H_2_O_2_ alone).

Consistent with the ROS results, the DNA damage assessed using the Comet assay was increased in the H_2_O_2_-exposed H9c2 cells (Figures 3(b) and 3(c)). Specifically, senescent cells demonstrated moderate/high DNA damage (corresponding to classes 3, 4, and 5) compared to control cells (*p* < 0.5, *p* < 0.1, and *p* < 0.001, Figure 3(c)). Notably, in the Nar-treated senescent cells, the reduction in the high numbers of cells with moderate/high DNA damage (classes 3, 4, and 5, *p* < 0.1, *p* < 0.001, and *p* < 0.1 versus the H_2_O_2_-treated cells, resp.) suggested a protective activity of Nar against ROS-induced genotoxic damage (Figure 3(c)).

It was indirectly shown that the reduction of both ROS and DNA damage is a result of Nar effect on antioxidative enzyme activity or by scavenger function. These results are consistent with previous studies that were carried out using the skin of hairless mice in which UVB irradiation was used to induce oxidative stress and premature aging [60]. Nar has been shown to mitigate UVB irradiation-induced oxidative stress by reducing superoxide anion production and the mRNA expression of a subunit of NADPH oxidase [60].

### 3.3. Nar Modulated the Mitochondrial Metabolic Activity and Increased the Calcium Retention Capacity

Additional cellular mechanisms that are often impaired during aging processes include those related to mitochondrial homeostasis [61–63]. Cellular senescence and mitochondrial dysfunction are hallmarks of aging; recent works have shown that mitochondrial defects can even cause a distinct senescence phenotype termed mitochondrial dysfunction-associated senescence [62, 63]. The roles of the mitochondrial electron transport chain, bioenergetic balance, redox state, metabolic signature, and calcium homeostasis have been recently studied in the context of the control of cellular growth arrest that is associated with senescence. These studies indicated that multiple mitochondrial signaling pathways in addition to mitochondrial ROS can induce cellular senescence [61]. Perturbation of mitochondrial homeostasis promotes the establishment and maintenance of cellular senescence, and various strategies to improve mitochondrial function have emerged as therapeutic approaches to aging [64].

**Figure 4:**
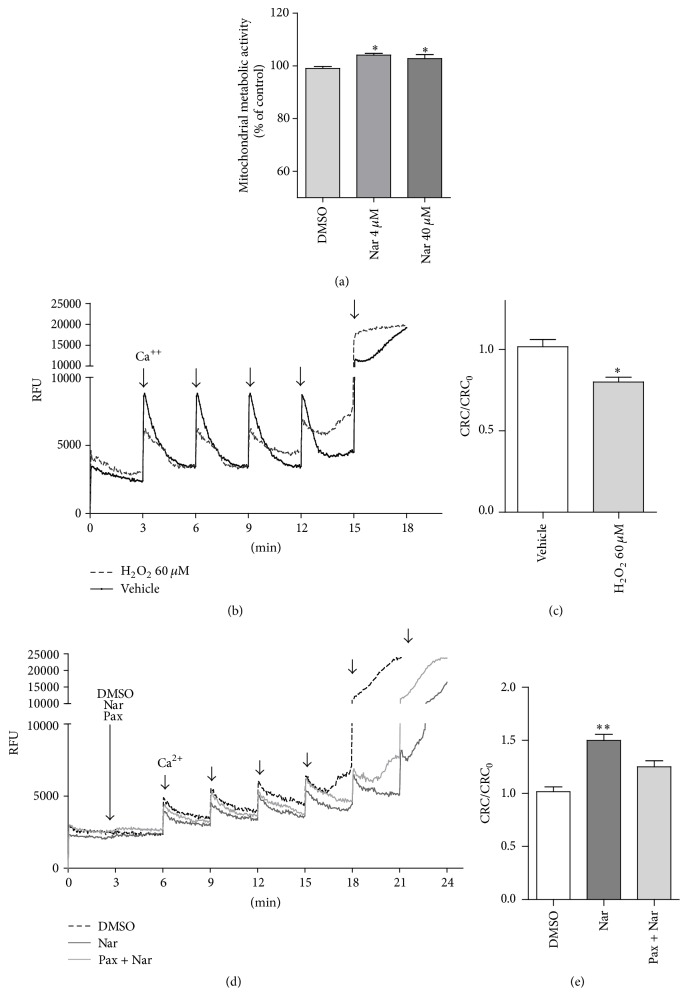
Effects of H_2_O_2_ and Nar on the mitochondrial oxidative metabolism activity and CRC. (a) H9c2 cells were treated with H_2_O_2_ and DMSO or Nar, and the mitochondrial oxidative metabolism activity was examined. (b–e) The cells were suspended in CRC medium and permeabilized with digitonin. To these cells, 0.25 M Calcium Green-5N and 5 mM succinate were added. This addition was followed by a series of Ca^2+^ pulses (10 nmoles) at 3-minute intervals until onset of the permeability transition (plateau). The Relative Fluorescence Unit (RFU) was recorded by spectrophotometer technique. ^*∗*^*p* < 0.05 versus DMSO-treated cells. (b) Representative traces of the H9c2 cell treatments with H_2_O_2_ and vehicle are shown in dashed black and gray, respectively. (c) The results from the H_2_O_2_-treated cells are shown as the CRC normalized to the CRC of control (CRC_0_). The data shown are the means ± SEM of three independent experiments. ^*∗*^*p* < 0.05 versus vehicle. (d) Representative traces of senescent H9c2 cells cotreated with DMSO (dashed black), Nar (dark gray), and Nar + Pax (light gray). (e) The results from senescent cells treated with Nar and Nar + Pax are shown as the CRC normalized to the CRC of control (CRC_0_). The data shown are the means ± SEM of three independent experiments. ^*∗∗*^*p* < 0.01 versus DMSO-treated cells.

In this context, the mitochondrial metabolic activity was explored in the Nar-treated H9c2 cells using a tetrazolium dye. The results demonstrated that Nar treatment increased the mitochondrial metabolic activity of senescent cells relative to that of the controls (*p* < 0.05, Figure 4(a)). Interestingly, parallel experiments in naïve H9c2 cells (not senescent) showed that Nar did not modulate mitochondrial metabolism per se, and it did not affect the number of senescent cells relative to the control (data not shown).

Next, the effects of Nar on a mitochondrial parameter recognized to be an indicator of damage resistance, the mitochondrial Ca^2+^-buffering cell capacity, were assessed. In particular, the mitochondrial calcium retention capacity, CRC, was explored; the upper limit of the CRC reflects the opening of the permeability transition pore (MPTP) and the dissipation of mitochondrial potential [65]. As shown in Figure 4, the cells treated with H_2_O_2_ (dashed black line) showed a significantly reduced ability to retain Ca^2+^ as indicated by the decrease in the CRC/CRC_0_ ratio (*p* < 0.05) (Figures 4(b) and 4(c)). Cells that had previously been treated with H_2_O_2_ were used to investigate the CRC in the presence of Nar. Nar was able to increase the mitochondrial CRC compared to the control cells (Figure 4(d), gray line versus dashed black line, Figure 4(e), *p* < 0.01).

The present data indicated that senescent H9c2 cells were more susceptible to the opening of the MPTP, in accordance with previous studies on cardiomyocytes isolated from old rats [66]. Notably, treatment of the cells with Nar was able to reduce this altered mitochondrial sensitivity.

It has been previously demonstrated that Nar is able to activate the mitoBK_Ca_ channels in mitochondria isolated from young rat hearts. This produces a mild depolarization of the membrane potential. This depolarization is responsible for reducing the uptake of Ca^2+^ into the mitochondrial matrix and preserving the mitochondria from the Ca^2+^ overload and the subsequent MPTP opening [4, 14]. To evaluate the involvement of mitoBK_Ca_ channels in the Nar-mediated CRC effects, the selective mitoBK_Ca_ blocker paxilline was tested in the CRC assessment. Paxilline alone had no significant effect on the CRC (data not shown), which might indicate that mitoBK_Ca_ channels were inactive under the resting condition. Interestingly, consistent with the modulation of mitoBK_Ca_ channels by Nar [14], the CRC increase exerted by Nar was partially prevented by cotreatment of the cells with paxilline (Figures 4(d), light gray line, and 4(e)). These results indicated that the Nar effects on the CRC might be mediated, at least in part, by the activation of mitoBK_Ca_ channels. Interestingly, it has been previously shown that the opening of mitoBK_Ca_ channels improves the function and energetic performance of cardiac mitochondria, which is consistent with our results [67]. One possible explanation may be the mild depolarization that occurs during the mitoBK_Ca_ channel opening. Indeed, it has been demonstrated that mice produce fewer free radicals and live longer when they have more uncoupled mitochondria, which show a low membrane potential, in muscles [68].

### 3.4. Nar Modulated the Levels of Estradiol Released by Cells and the Estrogen-Regulated Gene Expression

It is well known that estrogen is strongly implicated in cardiomyocyte survival and that its levels vary widely based on age [69, 70]. In general, premenopausal women are protected from coronary heart disease compared with age-matched men, but this female protection appears to be lost after menopause, which suggests that estrogen has beneficial effects on the cardiovascular system [22]. In addition to adrenal production of estrogen, the synthesis of estrogen by cardiac cells has been proposed, although this process has been very poorly elucidated (for a review, see [71]). Interestingly, some papers have suggested that local estrogen biosynthesis in the heart effectively activates the estrogen receptors *α* and *β* and downstream target genes in a gender-based fashion [72]. For these reasons, in the present study, the Nar effects on the estrogenic pathway were studied in senescent H9c2 cells. Specifically, we evaluated the estradiol released by cells and the estrogen-regulated gene expression.

**Figure 5:**
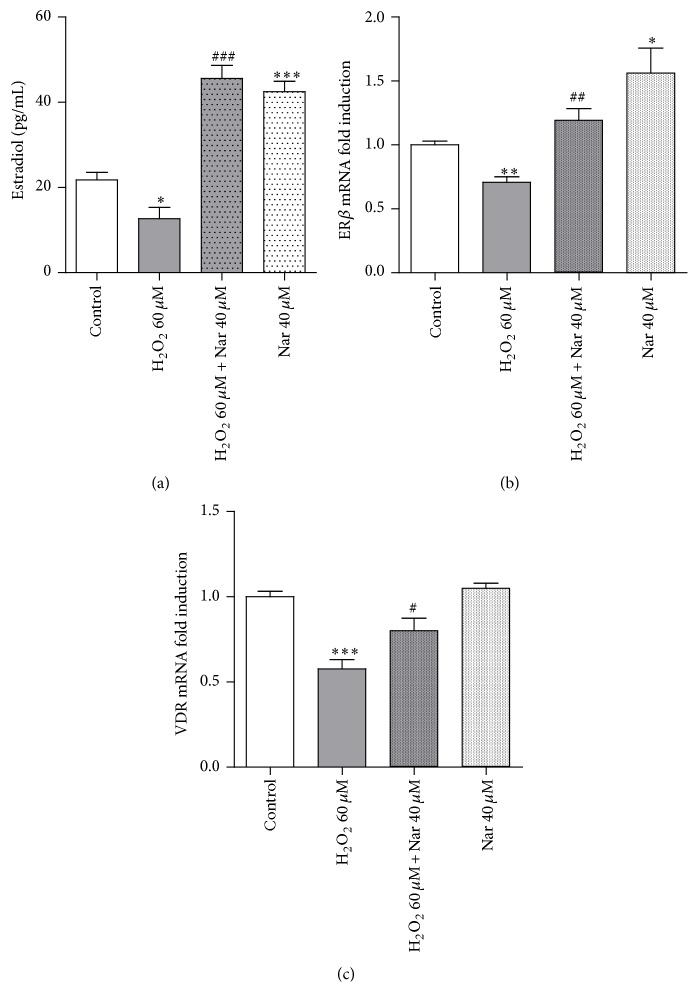
Effect of Nar on the levels of released estradiol and estrogen-regulated gene mRNA expression. (a) Each bar represents the mean ± SEM of three replicates from three independent experiments. The measured estradiol levels were within the reference range [24]. Specific calibration curves and appropriate positive/negative controls were carried out during the assay. ^*∗*^*p* < 0.05 and ^*∗∗∗*^*p* < 0.001 versus the control; ^###^*p* < 0.001 versus H_2_O_2_-treated cells. (b) ER *β* and (c) VDR mRNA expression as indicators of estrogenic activity in the senescent H9c2 cells. Each bar represents the mean ± SEM of three replicates from three independent experiments. ^*∗*^*p* < 0.05, ^*∗∗*^*p* < 0.01, and ^*∗∗∗*^*p* < 0.001 versus the control; ^#^*p* < 0.05 and ^##^*p* < 0.01 versus H_2_O_2_-treated cells.

As shown in Figure 5, H_2_O_2_ challenge induced a significant decrease in the levels of estradiol released by the cells (*p* < 0.05). This reduction was significantly counteracted by Nar treatment (*p* < 0.001 versus the H_2_O_2_-treated cells) (Figure 5(a)). Surprisingly, the level of estradiol in Nar-control cells (cells without H_2_O_2_ challenge) was higher than in the control cells (*p* < 0.001 versus control). It is reasonable to presume that such an increase may be due to the activation of cellular response mechanisms during the Nar treatment: one possible explanation may be that the response is related to cellular self-adaptive responses. Although the estrogenic potency of Nar has been controversial [7, 73], some studies have demonstrated the estrogenic effects of Nar and Nar-type flavonoids in mammalian in vitro systems using the estrogen-inducible MVLN luciferase assay [74].

Furthermore, the Nar-mediated expression of some commonly used parameters of drug estrogenic activity, specifically the estrogen-regulated genes ER *β* and VDR, was investigated. Quantitative real-time PCR experiments demonstrated that the expression of the ER*β* and VDR mRNA was significantly reduced in H_2_O_2_-treated cells (*p* < 0.01, Figure 5(b)) and that Nar was able to prevent this decrease (*p* < 0.01 versus the H_2_O_2_-treated cells). Consistent with the estradiol results, mRNA expression of both of these genes was increased by the Nar-only treatment.

Together, these data suggested that the ability of Nar to counterbalance the effects of H_2_O_2_ on the estrogenic pathway may contribute to its antisenescence effects.

## 4. Conclusions

In conclusion, Nar treatment of H9c2 cells prevented the prematurely induced senescence; Nar protection may involve multiple cellular pathways on the basis that it modulated the ROS levels, the mitochondrial potassium channels, and the estrogen-related pathways. In particular, the specific pathways involved seem to be those associated with the mitochondria. These results pave the way to exciting nutraceutical advances.

## Figures and Tables

**Table 1:** Nucleotide sequences, annealing temperature, and product size of the primers utilized in real-time RT-PCR experiments.

Gene	Primer nucleotide sequences	Annealing temperature (°C)	Product size (base pairs)
p16	FOR 5′- CCGAGAGGAAGGCGAACTC -3′	66.3	76
	REV 5′- GCTGCCCTGGCTAGTCTATCTG -3′	66.2	

p21	FOR 5′- GAGCAAAGTATGCCGTCGTC -3′	64.7	127
	REV 5′- CTCAGTGGCGAAGTCAAAGTTC -3′	65.0	

ER *β*	FOR 5′- CTACAGAGAGATGGTCAAAAGTGGA -3′	64.4	218
	REV 5′- GGGCAAGGAGACAGAAAGTAAGT -3′	63.6	

VDR	FOR 5′- GTGACTTTGACCGGAACGTG -3′	65.6	280
	REV 5′- ATCATCTCCCTCTTACGCTG -3′	60.8	
